# Genome-wide inventory and functional characterization of cannabinoid oxidocyclases in *Cannabis sativa*

**DOI:** 10.1093/hr/uhag023

**Published:** 2026-02-09

**Authors:** Minxuan Li, Sen Cai, Xu Bai, Yuzeng Ouyang, Lina Chen, Zhiyuan Zhang, Weiqi Tang, Marc Xu, Deyong Ao, Yaxing Li, Yunwei Sun, Liette Vasseur, Shijun You, Yang Liu, Nirit Bernstein, Yong Wang, Yuanyuan Liu

**Affiliations:** Haixia Institute of Science and Technology, State Key Laboratory of Agricultural and Forestry Biosecurity, Fujian Provincial Key Laboratory of Haixia Plant Systems Biology, Institute of Applied Ecology, Instrumental Analysis Center, Fujian Agriculture and Forestry University, Fuzhou 350002, China; State Key Laboratory of Plant Environmental Resilience, College of Life Science, Zhejiang University, Hangzhou, Zhejiang 310058, China; Haixia Institute of Science and Technology, State Key Laboratory of Agricultural and Forestry Biosecurity, Fujian Provincial Key Laboratory of Haixia Plant Systems Biology, Institute of Applied Ecology, Instrumental Analysis Center, Fujian Agriculture and Forestry University, Fuzhou 350002, China; Haixia Institute of Science and Technology, State Key Laboratory of Agricultural and Forestry Biosecurity, Fujian Provincial Key Laboratory of Haixia Plant Systems Biology, Institute of Applied Ecology, Instrumental Analysis Center, Fujian Agriculture and Forestry University, Fuzhou 350002, China; Haixia Institute of Science and Technology, State Key Laboratory of Agricultural and Forestry Biosecurity, Fujian Provincial Key Laboratory of Haixia Plant Systems Biology, Institute of Applied Ecology, Instrumental Analysis Center, Fujian Agriculture and Forestry University, Fuzhou 350002, China; Department of Plant Physiology, Swammerdam Institute for Life Sciences, University of Amsterdam, Science Park 904, Amsterdam 1098 XH, The Netherlands; Haixia Institute of Science and Technology, State Key Laboratory of Agricultural and Forestry Biosecurity, Fujian Provincial Key Laboratory of Haixia Plant Systems Biology, Institute of Applied Ecology, Instrumental Analysis Center, Fujian Agriculture and Forestry University, Fuzhou 350002, China; Haixia Institute of Science and Technology, State Key Laboratory of Agricultural and Forestry Biosecurity, Fujian Provincial Key Laboratory of Haixia Plant Systems Biology, Institute of Applied Ecology, Instrumental Analysis Center, Fujian Agriculture and Forestry University, Fuzhou 350002, China; Marine and Agricultural Biotechnology Laboratory, Institute of Oceanography, Minjiang University, Fuzhou 350108, China; Shenzhen Institutes of Advanced Technology, Chinese Academy of Science, Shenzhen 518055, China; Haixia Institute of Science and Technology, State Key Laboratory of Agricultural and Forestry Biosecurity, Fujian Provincial Key Laboratory of Haixia Plant Systems Biology, Institute of Applied Ecology, Instrumental Analysis Center, Fujian Agriculture and Forestry University, Fuzhou 350002, China; Haixia Institute of Science and Technology, State Key Laboratory of Agricultural and Forestry Biosecurity, Fujian Provincial Key Laboratory of Haixia Plant Systems Biology, Institute of Applied Ecology, Instrumental Analysis Center, Fujian Agriculture and Forestry University, Fuzhou 350002, China; Key Laboratory of Synthetic Biology, Center for Excellence in Molecular Plant Science, Institute of Plant Physiology and Ecology, Chinese Academy of Sciences, Shanghai 200032, China; Department of Biological Sciences, Brock University, St. Catharines, ON L2S 3A1, Canada; Haixia Institute of Science and Technology, State Key Laboratory of Agricultural and Forestry Biosecurity, Fujian Provincial Key Laboratory of Haixia Plant Systems Biology, Institute of Applied Ecology, Instrumental Analysis Center, Fujian Agriculture and Forestry University, Fuzhou 350002, China; Laboratory of Southern Subtropical Plant Diversity, Fairy Lake Botanical Garden, Shenzhen & Chinese Academy of Sciences, Shenzhen 518004, China; State Key Laboratory of Agricultural Genomics, BGI-Shenzhen, Shenzhen 518083, China; Institute of Soil Water and Environmental Science, Volcani Institute, 68 HaMaccabim Road, PO Box 15159, Rishon LeZion 7505101, Israel; Key Laboratory of Synthetic Biology, Center for Excellence in Molecular Plant Science, Institute of Plant Physiology and Ecology, Chinese Academy of Sciences, Shanghai 200032, China; Haixia Institute of Science and Technology, State Key Laboratory of Agricultural and Forestry Biosecurity, Fujian Provincial Key Laboratory of Haixia Plant Systems Biology, Institute of Applied Ecology, Instrumental Analysis Center, Fujian Agriculture and Forestry University, Fuzhou 350002, China

*Cannabis sativa* is distinguished by its production of cannabinoids that exhibit diverse therapeutic effects, including antiemetic, antispasticity, anxiolytic, and antiepileptic activities. Over 120 phytocannabinoids have been identified, with Δ^9^-tetrahydrocannabinol (THC) and cannabidiol (CBD) being the most abundant. THC is the principal psychoactive constituent responsible for the euphoric effects of cannabis, while CBD is nonpsychoactive and counteracts several adverse effects of THC. Understanding the biosynthesis of these compounds is critical due to their pharmacological potential.

Cannabinoid oxidocyclases (CBSs), a group of berberine bridge enzyme (BBE)-like proteins, catalyze the committed step in cannabinoid biosynthesis [1]. CBSs contain a flavin adenine dinucleotide (FAD)-binding domain and a substrate-binding BBE-like domain, which mediate the conversion of the substrate CBGA into acidic cannabinoids, including Δ^9^-tetrahydrocannabinolic acid (THCA), cannabidiolic acid (CBDA), and cannabichromenic acid (CBCA). These acidic cannabinoids are subsequently converted into their neutral forms (THC, CBD, and CBC) through non-enzymatic decarboxylation when exposed to heat [2]. The major cannabinoid synthases (THCAS, CBDAS, CBCAS) have been extensively characterized. Heterologous expression of *THCAS* in tobacco leaves produced THCA and CBCA. Expression of *THCAS* and *CBDAS* in *Pichia pastoris* yielded mixtures of THCA, CBDA, and CBCA and additional unknown compounds, while C*BCAS* expressed in *P. pastoris* catalyzes the specific formation of CBCA [3]. Protein crystallization and site-directed mutagenesis of THCAS revealed critical residues near substrate-binding pocket and FAD-binding site [1]. Nevertheless, additional CBSs contributing to cannabinoid diversity remain largely unexplored.

**Figure 1:**
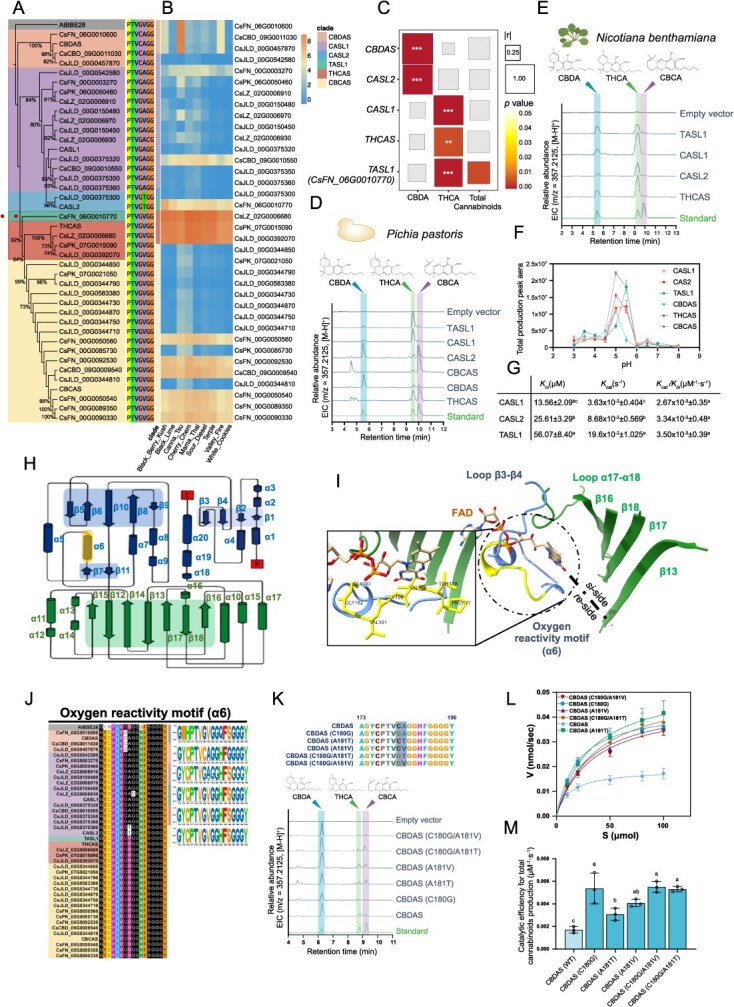
(A) Phylogenetic relationships among the CsCBSs. Thirty-six CsCBSs are clustered into five clades, namely CBDAS clade, CASL clade, THCAS clade, CBCAS clade , and an individual CsCBS, CsFN_06G0010770 (TASL1, pointed by arrow). AtBBE-like 28 is served as an outgroup. Sequence alignment of the reactive oxygen motif (PTVGVGG) is shown besides. (B) Expression profiles of the identified 36 *CsCBSs* in mature glandular trichomes of nine cannabis varieties. (C) Correlation analysis between expression level of five *CBSs* with cannabinoid contents (CBDA, THCA, and total cannabinoids) in 21 cannabis varieties. The *r* and *P-*values are based on the Spearman correlation analysis. ^*^P < 0.05; ^**^P < 0.01; ^***^P < 0.001. (D) *In vitro* enzymatic assays of TASL1, CASL1, CASL2, CBCAS, CBDAS, and THCAS proteins expressed by *P. pastoris*, using CBGA as substrate, pH 5.0. (E) Functional characterization of TASL1, CASL1, CASL2, and THCAS in tobacco, using CBGA as substrate. (F) Effect of pH on the activity of recombinant CBSs proteins expressed by *P. pastoris*. Data are displayed as mean ± SD, *n* = 3. (G) Kinetic parameters of CASL1, CASL2, and TASL1. Data are presented as mean ± SD, *n* = 3. Lowercase letters indicate statistically significant differences based on Tukey’s multiple-range test (*P* < 0.05). (H) Topology diagram of CsCBSs. α-helices are shown as cylinders and β-strands as arrows. The flavin-binding motif (located within β12-β18), the substrate-binding motif (β1-β10), and the oxygen reactivity motif (α6) are shaded. (I) 3D structure of CsCBSs active pocket. Inset figure illustrates the residues forming the oxygen reactivity motif in THCAS (PDB: 3vte), shown in a stick model. (J) Multiple sequence alignment of the oxygen reactivity motif and comparison of the sequence logos of five CsCBS subclades and AtBBE-like 28. (K) Enzymatic reaction product profile of CBDAS and its variants. Mutation sites are highlighted. (L) Michaelis–Menten kinetics of CBDAS and CBDAS variants. CBGA in different concentrations was used as substrate and the production efficiencies of total cannabinoids were monitored. Data are presented as mean ± SD and each repeat is displayed as hollow circle, *n* = 3. (M) *In vitro* catalytic efficiency of CBDAS and CBDAS variants for total cannabinoids production expressed by *P. pastoris*. Data are presented as mean ± SD, *n* = 3. Lowercase letters above each column of CBDAS and CBDAS variants indicate significant differences by Tukey’s multiple-range test (*P* < 0.05).

To address this, we performed a genome-wide survey of five cannabis genomes and identified 36 putatively functional *CsCBS* genes. These included 8 from CSFN, 4 from CSPK, 3 from CSCBD, and 17 from CSJLD, plus five previously reported genes (Fig. 1A). Phylogenetic analysis grouped these sequences into five clades, largely corresponding to functionally characterized CBSs, except one clade harboring *CBDAS-like* genes (*CBDAS-like 1*, *CASL1*, and *CBDAS-like 2 CASL2*) of unknown function [4]. A distinct gene, *CsFN_06G0010770*, that diverged from other CBSs, was named *THCAS-like 1* (*TASL1*). Transcriptome profiling of mature glandular trichomes across nine varieties revealed high expression of *CBDAS*, *THCAS*, and *CBCAS*, as well as detectable expression of *CASL1*, *CASL2*, and *TASL1* (Fig. 1B) [2]. Expression correlation analyses across 21 female flower varieties showed that *CBDAS* and *THCAS* expression levels correlated with CBDA and THCA accumulation, respectively. Interestingly, *TASL1* and *CASL1* expression correlated with THCA production, whereas *CASL2* correlated with CBDA (Fig. 1C and Fig. S1), suggesting these enzymes might function differently *in planta*. Heterologous expression of *CASL1*, *CASL2*, and *TASL1* in *P. pastoris* and *Nicotiana benthamiana* confirmed their enzymatic activity, while revealing distinct product preferences (Fig. 1D and E). For the *N. benthamiana* assays, *Agrobacterium tumefaciens* GV3101 harboring pEAQ-CASL1/CASL2/TASL1 was cultured and used for agroinfiltration (10 mM MES, 10 mM MgCl_2_, 100 μM acetosyringone to OD_600_ = 0.6), the CBGA substrate was infiltrated into the same leaf areas at 4 days post-agroinfiltration, and leaf tissues were harvested for metabolic analysis 1 day later. CASL1 and TASL1 converted CBGA to THCA as the major product in both heterologous systems, whereas CASL2 exhibited host-dependent activity, producing CBDA and CBCA as the predominant products in *P. pastoris*, but primarily THCA in *N. benthamiana*. All three enzymes displayed a pH optimum ~5.5, consistent with apoplastic conditions reported for CBS activity (Fig. 1F) [5]. Kinetic analyses indicated that TASL1 and CASL2 exhibited comparable catalytic efficiencies (*k*cat/*K*m), both of which were modestly higher than that of CASL1 (Fig. 1G). The *K*_m_ value of TASL1 (56.07 on average) was much higher than CASL1 and CASL2 (13.56 and 25.61 on average, respectively), indicating a lower affinity between TASL1 and CBGA, while the *k*_cat_ value of TASL1 was about 5.4 and 2.2 times higher than that of CASL1 and CASL2, respectively, indicating a greater turnover efficiency of TASL1. Collectively, these results indicate that CASL1, CASL2, and TASL1 likely contribute differently to cannabinoid biosynthesis and overall cannabinoid yield.

To explore the basis of functional diversity among CsCBSs, we next examined structural motifs that may influence product profiles and catalytic efficiency. Overall, CsCBSs display a conserved topology comprising distinct FAD-binding and substrate-binding domains (Fig. 1H). Four β-sheets (β13, β16, β17, and β18) and the loop connecting α17 and α18 form a wide-open substrate-binding pocket located on the *si*-side of the FAD isoalloxazine ring (Fig. 1I). The loop connecting β3 to β4 and an α-helix (α6) located on the *re*-side of the FAD isoalloxazine ring. A motif containing α6 and its flanking amino acids was previously identified as an oxygen reactivity motif (ORM), which may function as an oxygen cavity that traps dioxygen and catalyze the oxidation of the flavin [5].

We next compared sequence homology across seven conserved motifs forming the active site of BBE-like enzymes in *Arabidopsis* (Fig. S2). Two residues within the ORM were conserved within specific CsCBS phylogenetic clades (Fig. 1J), and their variation paralleled the overall phylogenetic relationships among family members (Fig. 1A). In THCAS, FAD-mediated redox catalysis involves hydride transfer from CBGA to the oxidized FAD cofactor [1], while the ORM facilitates reoxidation of reduced FAD for subsequent catalytic cycles [5]. To assess the contribution of individual ORM residues to CBS activity, we introduced targeted substitutions in CBDAS: C180G and A181T or A181V. All variants retained CBDA as the main product (Fig. 1K) but exhibited increased catalytic efficiency (*k*_cat_/*K*_m_) relative to the wild type (Fig. 1L and M). Notably, the C180G substitution, which likely enlarges the oxygen-binding cavity, led to a 3-fold increase in catalytic efficiency, while A181V conferred greater activity than A181T (Fig. 1M), indicating that amino acid substitutions within the ORM can significantly enhance catalytic efficiency in cannabinoid biosynthesis. Molecular docking of CBDAS and its variants with CBGA as the ligand revealed that the variants display higher binding affinities for CBGA than the wild-type enzyme. Within the active pocket of the CBDAS variants, the CBGA substrate is positioned in closer proximity to the FAD cofactor and the essential active site residues TYR483 (Fig. S3), providing a structural basis for the enhanced catalytic performance.

In summary, our genome-wide analysis identified 36 putatively functional *CsCBS* genes, including the evolutionarily distinct *TASL1*. Transcriptomic and biochemical evidence indicates TASL1, CASL1, and CASL2 contribute to cannabinoid diversity, with TASL1 catalyzing CBGA conversion to THCA, CBDA, and CBCA. Structural and mutational analyses reveal that ORM residues influence catalytic efficiency, reflecting evolutionary adaptation toward optimized cannabinoid production. These findings advance our understanding of the genetic and enzymatic framework underlying cannabinoid formation and provide a basis for enzyme engineering, metabolic pathway optimization in plants, and applications in synthetic biology.

## Data Availability

The raw sequence data and genome assembly used for this project are available from CNCB BioProject (China National Center for Bioinformation Biological Project Library: https://ngdc.cncb.ac.cn/bioproject/) under accession number PRJCA015041. The genome assembly for *C. sativa* ‘CSLZ’ have been deposited at CNCB GWH (China National Center for Bioinformation Genome Warehouse: https://ngdc.cncb.ac.cn/gwh/) under Bioproject codes GWHBWDA00000000. The raw data of genome sequencing have been deposited in the CNCB GSA (China National Center for Bioinformation Genome Sequence Archive: https://ngdc.cncb.ac.cn/gsa/) database under accession number: CRA009905. The raw data of transcriptome sequencing comes from SRP168446 in the NCBI SRA database. The supplementary tables and figures supporting the conclusions of this article are available on the Figshare database under the accession https://doi.org/10.6084/m9.figshare.30919262.
